# Development of a biodegradable antifibrotic local drug delivery system for glaucoma microstents

**DOI:** 10.1042/BSR20180628

**Published:** 2018-08-31

**Authors:** Thomas Stahnke, Stefan Siewert, Thomas Reske, Wolfram Schmidt, Klaus-Peter Schmitz, Niels Grabow, Rudolf F. Guthoff, Andreas Wree

**Affiliations:** 1 Department of Ophthalmology, Rostock University Medical Center, Doberaner Str. 140, Rostock 18057, Germany; 2 Institute for Implant Technology and Biomaterials e.V., Friedrich-Barnewitz-Straße 4, Rostock 18119, Germany; 3 Institute of Biomedical Engineering, Rostock University Medical Center, Friedrich-Barnewitz-Straße 4, Rostock 18119, Germany; 1 Institute of Anatomy, Rostock University Medical Center, Gertrudenstraße 9, Rostock 18057, Germany

**Keywords:** caffeic acid phenethyl ester, fibrosis, glaucoma, microstent, paclitaxel, pirfenidone

## Abstract

To prevent implant failure due to fibrosis is a major objective in glaucoma research. The present study investigated the antifibrotic effects of paclitaxel (PTX), caffeic acid phenethyl ester (CAPE), and pirfenidone (PFD) coated microstent test specimens in a rat model. Test specimens based on a biodegradable blend of poly(4-hydroxybutyrate) biopolymer and atactic poly(3-hydroxybutyrate) (at.P(3HB)) were manufactured, equipped with local drug delivery (LDD) coatings, and implanted in the subcutaneous white fat depot. Postoperatively, test specimens were explanted and analyzed for residual drug content. Fat depots including the test specimens were histologically analyzed. *In vitro* drug release studies revealed an initial burst for LDD devices. *In vivo*, slow drug release of PTX was found, whereas it already completed 1 week postoperatively for CAPE and PFD LDD devices. Histological examinations revealed a massive cell infiltration in the periphery of the test specimens. Compact fibrotic capsules around the LDD devices were detectable at 4–36 weeks and least pronounced around PFD-coated specimens. Capsules stained positive for extracellular matrix (ECM) components. The presented model offers possibilities to investigate release kinetics and the antifibrotic potential of drugs *in vivo* as well as the identification of more effective agents for a novel generation of drug-eluting glaucoma microstents.

## Introduction

Glaucoma is a leading cause of irreversible blindness worldwide and is mostly characterized by an increased intraocular pressure (IOP), which results from disturbances in the outflow of aqueous humor from the eye. Permanently increased pressure conditions lead to damage of the retinal ganglion cells and degeneration of the optic nerve fibers, resulting in a loss of vision and finally blindness, if untreated [1]. Until now, reduction in IOP is the only available treatment to prevent nervous degeneration [2,3]. Medicinal therapy represents the first-line treatment for IOP lowering [4,5]. In case of insufficient IOP reduction by means of medicinal therapy, surgical interventions have been used to drain aqueous humor from the anterior chamber of the eye into an extraocular compartment. Trabeculectomy and deep sclerectomy [6–8] have been used most commonly. Additionally, cyclophotocoagulation [9,10] provides an alternative therapy to lower IOP by decreasing the aqueous humor production.

As a further approach to conventional surgical interventions, alloplastic devices for the drainage of aqueous humor from the anterior chamber into different outflow areas, particularly into Tenon’s capsule, have been used [11]. Clinically used implants in a tube and plate design are the Molteno (Molteno Ophthalmic Ltd., Dunedin, New Zealand), the BAERVELDT (Abbott Medical Optics Inc., Santa Ana, CA, U.S.A.), and the Ahmed (New World Medical Inc., Rancho Cucamonga, CA, U.S.A.) drainage devices. These implants differ in terms of material and design, and the Ahmed implant has a flow-restricting valve mechanism [12]. Other approaches prefer the suprachoroidal space as outflow area to lower IOP [13–15]. The disadvantages of surgical interventions as well as implantations of drainage devices are the fibrotic and uncontrollable scarring processes, which can lead to a failure of the newly created draining channels or to an implant occlusion [16] and as a consequence to an IOP increase. Thus, the development of an antifibrotic drug-eluting microstent represents a major objective for improving the clinical success rates of glaucoma drainage devices (GDD).

Fibrotic and scarring processes are mainly driven by the proliferation of fibroblasts and exuberant extracellular matrix (ECM) expression. It was shown that fibroblasts from different ocular tissues differ in their mRNA profiles [17] and in their expression of ECM components [18]. The retro-orbital white fat depot (corpus adiposum orbitae) with probably a different fibrotic behavior and low pressure values of 4–14 mmHg [19–21] has been discussed as a possible drainage area. The advantage of the retro-orbital white fat depot for drug-eluting drainage devices is its distance from the very sensitive structures of the eye, like the retina. Cinti [22] demonstrated in his work that in rodents the subcutaneous white fat depots anterior to the hind legs are metabolically much less active compared with the very active brown fat areas, which makes these regions comparable with the corpus adiposum orbitae in humans and thus a suitable model to analyze postoperative reactions.

To address the problem of fibrosis, alloplastic drainage devices can be coated with antifibrotic drugs as it is done with drug-eluting stents in cardiovascular treatment [23,24]. The most commonly used antifibrotic agents are the cytostatics mitomycin C and 5-fluorouracil [25], which inhibit mitotic processes. Additionally, the potent inhibitor of mitosis, paclitaxel (PTX) [26,27], was studied in this context. Some disadvantages of these cytostatics are unspecific influences and toxic effects on all cell types as a result of their mechanisms of action. For side effect minimization, specific drugs/agents have to be identified, which only act on fibrotic relevant fibroblasts and their synthesis of ECM components without impairing cell and tissue viability. In this context, caffeic acid phenethyl ester (CAPE) is one of the most interesting bioactive compounds. CAPE was shown to be an effective agent to prevent fibrotic processes in pulmonary fibrosis by influencing some profibrotic/antifibrotic key mediators including TGF-β1, TNF-α and prostaglandin E2 [28], and the synthesis of collagen I [29]. Also its antioxidant activity and modulatory impact on the immune system has been described [30].

Another specific antifibrotic agent is pirfenidone (PFD), which has been shown to decrease the expression of fibrosis relevant cytokines (TGF-β1–3) [31] and ECM components [32]. PFD has also been used as an oral formulation for systemic treatment of idiopathic pulmonary fibrosis [33]. PFD can also modulate immune responses as demonstrated in an abdominal adhesion rat model [34].

**Figure 1:**
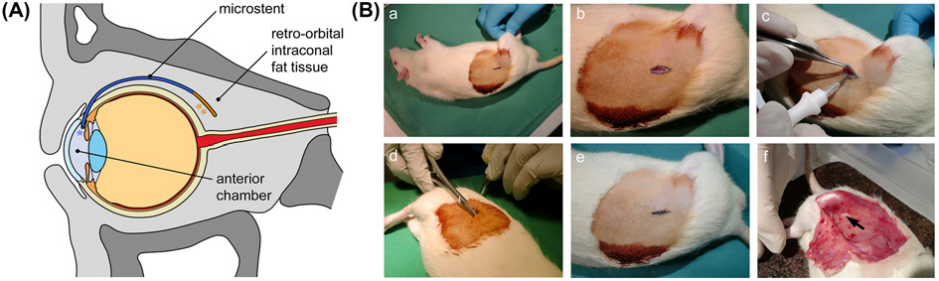
Illustration of the microstent concept and photo documentation of the minimally invasive surgical intervention (**A**) Schematic drawing of a microstent for the drainage of aqueous humor from the anterior chamber of the eye into the retro-orbital intraconal fatty tissue; microstent with micro-mechanical valve for the prevention of hypotony (*) located in the anterior chamber and LDD coating (**) for the prevention of fibrosis located in the outflow area. (**B**) Implantation of test specimen into the subcutaneous white fat depot of rats and explantation procedure. (a) Shaved and disinfected implantation area with mark for incision. (b) Carefully opened cutis. (c) Test specimen injection into white fat depot using a PICO-ID-Chip-Injector. (d) Wound closure by suture. (e) With two stitches closed cutis. (f) Explantation of test specimen. Arrow marks the implant.

In the present study, a local drug delivery (LDD) coating for a novel biodegradable glaucoma microstent was developed and analyzed *in vitro* and *in vivo* in terms of drug release behavior and tissue response to address the postoperative problem of scarring and fibrosis around the stent in the outflow area. The concept of our implant-based regenerative approach for the drainage of aqueous humor into the retro-orbital intraconal fat tissue is shown in Figure 1A.

As an alternative to conventional permanent, biostable GDDs, the presented biodegradabale microstent serves as temporary pathway for controlled drainage of aqueous humor. Our initial experiments using these biodegradable polymers as GDD provided promising results [35]. An LDD coating in the outflow area was designed to prevent fibrosis in the postoperative period. After microstent degradation, the remnant channel should allow for long-term effective drainage without the complications associated with foreign-body reactions to the biomaterial or mechanical irritation, often observed in cases of permanent GDD.

The aim of the current study was the evaluation of drug release and antifibrotic effects of different LDD coatings, containing the drugs PTX and the more specific agents CAPE and PFD, in a rat model. Therefore, GDD test specimens with different LDD coatings were implanted subcutaneously into the white fat depots in front of the right hind leg of rats. After explantation at different postoperative points in time, tissue samples including the test specimens were analyzed with regard to residual drug-loading and fibrotic responses.

## Materials and methods

### Manufacturing and characterization of test specimens

Test specimens were composed of tubing and an LDD coating, both manufactured based on biodegradable polymeric materials. A polymer blend from poly(4-hydroxybutyrate) (P(4HB); P4HB biopolymer, Tepha, Inc., Lexington, MA, U.S.A.), and amorphous atactic poly(3-hydroxybutyrate) (at.P(3HB)) in a blend ratio of 50/50% (w/w) was used to prepare the tubing and the coatings. Synthesis of at.P(3HB) was conducted according to Jedlinski et al. [36] and Hubbs et al. [37] by ring-opening polymerization of β-butyrolactone using potassium acetate as a catalyst [38]. Three different LDD coatings were based on a homogeneous mixture of the polymer blend and the drug PTX (Cfm Oskar Tropitzsch e.K., Marktredwitz, Germany), CAPE (Sigma–Aldrich Corp., St. Louis, MO, U.S.A.), or PFD (Sigma–Aldrich Corp., St. Louis, MO, U.S.A.) in a mixing ratio of 85/15% (w/w), respectively. In a control group, tubing without LDD coating was used.

Tubing with a wall thickness of 75 µm was manufactured in a semiautomatic process using a dip-coating robot (KSV NIMA Dip Coater, Biolin Scientific Holding AB, Stockholm, Sweden). Stainless steel mandrels (diameter: 300 µm, length: 60 mm) were dipped repeatedly into the polymer solution prepared from 1150 mg chloroform (Sigma–Aldrich Corp., St. Louis, MO, U.S.A.) and 100 mg of the polymer blend P(4HB)/at.P(3HB) 50/50% (w/w). A withdrawal speed of 300 mm.min^−1^ was used. After each repetition, the mandrels were dried for 20 min at ambient temperature and rotated at 180°. Tubing diameter was measured in 0.5 mm increments along the longitudinal axis using a biaxial laser scanner (ODAC 32 XY, Zumbach Electronic AG, Orpund, Switzerland) after the mandrels were removed.

The LDD coatings of the tubular test specimens (length: 10 mm) were applied with an airbrush process using a polymer solution prepared from 28.5 g chloroform (Sigma–Aldrich Corp., St. Louis, MO, U.S.A.) and 100 mg of the polymer blend/drug mixture. A mass of 126 µg, corresponding to a drug loading of 1.4 µg.mm^−2^, was the desired nominal coating weight. Measurement of the coating mass was conducted using an ultramicrobalance (XP6U, Mettler-Toledo International, Inc., Greifensee, Switzerland).

After preparation, the test specimens were dried for 7 days in vacuum at ambient temperature. Test specimens from the control, the PTX and the CAPE groups were sterilized by means of ethylene oxide as described previously [35]. For PFD-coated test specimens, cooled β sterilization at −15°C in a nitrogen atmosphere was applied, using a radiation dose of 25 kGy in 4 min. Prior to β sterilization, test specimens were cooled at −40°C for 24 h.

Morphological analysis of the manufactured test specimens was performed using SEM (Quanta 250 FEG, FEI, Hillsboro, OR, U.S.A.) in environmental mode (ESEM) at a vacuum pressure of 0.5 mbar and an accelerating voltage of 5 kV.

### Drug release *in vitro*

Analysis of temporal drug release from the test specimens was performed *in vitro* in 2 ml of 0.9% saline solution at 37°C. To protect CAPE from oxidation, 0.2% of ascorbic acid (Sigma–Aldrich Corporation, St. Louis, MO, U.S.A.) was added to the saline solution.

During *in vitro* drug release studies, the test specimens were incubated on a rotating platform-shaking device (Unimax 1010, Heidolph Instruments GmbH & Co. KG, Schwabach, Germany) at 100 rpm. After a defined time of incubation *Δt_i_*, the saline solution was collected and drug content *m_i_ (Δt_i_)* was analyzed by means of HPLC (KNAUER Wissenschaftliche Geräte GmbH, Berlin, Germany). Prior to HPLC analysis, the saline extracts were diluted 1:1 (v/v) with methanol. Cumulative released drug mass m*_j_* after *j* cycles was calculated by summation of individual values.

After drug release stagnated, the residual drug in the test specimen was extracted by methanol and analyzed using HPLC. Individual test specimens additionally were taken after extraction and completely dissolved in chloroform again and analyzed using HPLC to find residual drug amounts. A summary of HPLC conditions used for analysis of the different drugs PTX, CAPE, and PFD is shown in Table 1.

**Table 1:** Summary of HPLC conditions used for *in vitro* drug release studies

	PTX	CAPE	PFD
Column	Chromolith fast gradient RP-18e 50-2 (Merck KGaA, Darmstadt, Germany)	Eurospher 100 C18, 5 μm, 125 × 4 mm ID (Knauer GmbH, Berlin, Germany)	Eurospher 100 C18, 5 μm, 125 × 4 mm ID (Knauer GmbH, Berlin, Germany)
Mobile phase	Acetonitrile/phosphate buffer solution (0.005 M, pH 3.5) (50/50% v/v)	Methanol/water (65/35% v/v)	Acetonitrile/water (27/73%) v/v with 0.2% acetic acid
Flow rate	0.3 ml.min^−1^	1.0 ml.min^−1^	1.0 ml.min^−1^
Column temperature	23°C	30°C	45°C
Detection wavelength (UV)	230 nm	323 nm	310 nm
Injected sample volume	20 µl	20 µl	20 µl
Retention time	3.0 min	4.5 min	6.0 min
Measurement range	0.1–20 µg.ml^−1^	0.1–20 µg.ml^−1^	0.1– 20 µg.ml^−1^

### Drug release *in vivo*

For the analysis of temporal drug release *in vivo*, test specimens explanted at different postoperative points in time *t_i_* were analyzed for residual drug loading *m_res_ (t_i_)* by means of HPLC. Prior to HPLC, the drugs were extracted from test specimens by methanol. Released drug mass *m_i_ (t_i_)* at the time *t_i_* was calculated by subtracting *m_res_ (t_i_)* from the initial drug loading *m_init_*.

### Ethical consent

All animal studies were approved by the local authorities (Landesamt für Landwirtschaft, Lebensmittelsicherheit und Fischerei Mecklenburg-Vorpommern (LALLF-MV)) and conducted in accordance with the German Animal Welfare Act (Approval ID: 7221.3-1.1-085/12).

### Animals

Adult male Wistar rats (strain Crl:WI BR, Charles River Wiga GmbH, Sulzfeld, Germany) aged approximately 3 months were used and individually housed in standard cages at 22 ± 2°C under a 12-h light/dark cycle in a specific pathogen-free housing, with free access to tap water and a standard diet.

### Surgical intervention

Rats weighing 280–300 g were anesthetized by intraperitoneal injection of ketamine (Bela-pharm GmbH, Vechta, Germany; 50 mg/kg body weight) and xylazine (Rompun, Bayer AG, Leverkusen, Germany; 4 mg/kg body weight). The implantation area was shaved and disinfected with Betaisodona (Mundipharma GmbH, Limburg, Germany). Cutis was carefully opened with a scalpel and by a minimally invasive surgical intervention, the test specimens were implanted into the subcutaneous white fat depots in front of the right hind leg using a PICO-ID-Chip-Injector (AgnTho’s, Lidingö, Schweden) (Figure 1B). This region was selected for the implantation because these white fat depots are metabolically less active compared with the active brown fat areas [22]. Therefore, the subcutaneous white fat of rats is comparable with the retrobulbar orbital white fat depot (corpus adiposum orbitae) in humans. At defined postoperative points in time (1, 2, 4, 12, and 36 weeks) animals were killed with an overdose of ketamine and xylazine. Test specimens were explanted from six animals per group: four for HPLC analysis of residual drug content. The remaining two specimens were explanted after the rats were perfused with 3.7% paraformaldehyde (PFA) dissolved in PBS (pH 7.4) prior to histological analysis. Fat depots were explanted, postfixed in 3.7% PFA for 24 h, and embedded in paraffin for histological investigation.

### Histology

Histological sections of 5 µm thicknesses were AZAN stained using a standard protocol [39]. Briefly, rehydrated sections were stained in filtered and preheated (56°C) 0.1% aqueous azocarmine G solution (Merck Millipore, Darmstadt, Germany) and transferred into 0.1% aniline in 96% ethanol (Merck Millipore) until cytoplasm, connective tissue, and nuclei were well defined. After staining with 0.25% Aniline Blue, 1% Orange G (Sigma–Aldrich, Taufkirchen, Germany; Merck) sections were dehydrated with absolute alcohol and xylene and mounted with Entellan (Merck Millipore).

As a second histological observation, sections were stained with Hematoxylin–Eosin (H&E) [39]. Shortly, after dewaxing in xylene and hydration in a decreasing alcoholic row, sections were stained with Hematoxylin (Merck Millipore), followed by alcoholic dehydration and 1% Eosin G (Merck Millipore) staining. Differentiation occurs in 90% alcohol followed by clearing in xylene and finally mounting in Entellan (Merck Millipore).

For detailed investigation of the fibrotic and inflammatory responses, immunohistochemistry using the avidin–biotin complex immunoperoxidase method (Vector Laboratories, Burlingame, CA, U.S.A.) was carried out as described before [40]. Briefly, paraffin slides were dewaxed, rehydrated, and non-specific protein binding was blocked with 10% BSA in TBS including 100 mM lysine and 1% Triton X-100 at room temperature for 60 min. After washing, sections were incubated with the primary antibodies overnight at 4°C. Following another wash cycle, sections were incubated with either biotinylated rabbit anti-goat IgG or with biotinylated horse anti-mouse IgG for 2 h at room temperature before color development with diaminobenzidine. For control sections, the primary antibody was omitted. After mounting with Entellan (Merck Millipore) sections were analyzed using a BX51 microscope (Olympus, Hamburg, Germany) and Stereoinvestigator 8.0 software.

The following primary and secondary antibodies were used for IHC staining (Table 2).

**Table 2:** Antibodies used in the present study

Reagent	Supplier	Catalog number
Mouse monoclonal anti-collagen I	Abcam (Cambridge, U.K.)	ab6308
Mouse monoclonal anti-collagen VI	Abcam (Cambridge, U.K.)	ab78504
Rabbit polyclonal fibronectin	DPC Biermann GmbH (Germany)	DP013
Mouse monoclonal fibronectin	Sigma–Aldrich (Germany)	F7387
Rabbit polyclonal CD11b	Abcam (Cambridge, U.K.)	Ab52478
Biotinylated anti-ms IgG (H+L)	Vector/Biozol (Eching, Germany)	VEC-BA-2000-CE
Biotinylated anti-ms IgM	Vector/Biozol (Eching, Germany)	VEC-BA-2020
Biotinylated anti-rb IgG (H+L)	Vector/Biozol (Eching, Germany)	VEC-BA-1000-CE

The datasets generated and analyzed during the current study are available from the corresponding author on reasonable request.

## Results

### Manufacturing and characterization of test specimens

**Figure 2:**
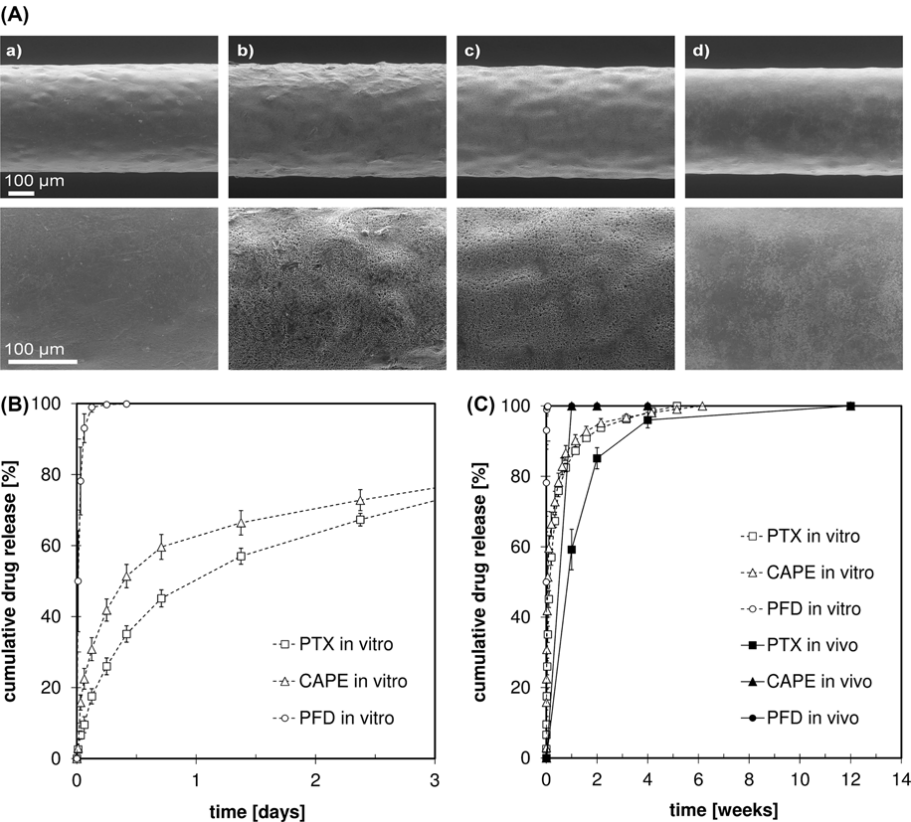
Manufactured test specimens and quantitation of drug release *in vitro* and *in vivo* (**A**) Representative scanning electron micrographs of test specimens from (a) group 1 (no LDD coating), (b) group 2 (PTX LDD coating), (c) group 3 (CAPE LDD coating), and (d) group 4 (PFD LDD coating) at 150× and 400× magnification, respectively. (**B**) Cumulative drug release from different LDD devices *in vitro* in an initial time frame of the release studies (each *n*=4, normalized to recovered drug mass). (**C**) Drug release from different LDD devices *in vitro* and *in vivo* within 12 weeks (each *n*=4).

Average wall thickness and drug mass of the manufactured test specimens are summarized in Table 3. Representative scanning electron microscope (SEM) images of the test specimens are shown in Figure 2A. Scanning electron micrographs showed smooth surfaces for all groups at low magnification (150×). Microporous surfaces of group 2 (PTX LDD coating) and group 3 (CAPE LDD coating) were visible at high magnification (400×).

**Table 3:** Average wall thickness and drug mass of manufactured test specimens: mean ± S.D. (each *n*=35)

Group	Tubing	LDD coating	
	Wall thickness [µm]	Drug	Drug mass [µg]
1	74.5 ± 9.0	-	-
2	70.9 ± 5.3	PTX	20.3 ± 1.4
3	65.8 ± 5.8	CAPE	19.5 ± 0.8
4	68.8 ± 5.1	PFD	19.8 ± 0.9

### Drug release *in vitro*

Drug release studies revealed a burst release followed by a more retarded release phase for all LDD devices. Overall drug recovery from *in vitro* studies of PTX, CAPE, and PFD LDD devices was 70.0 ± 6.5, 75.9 ± 0.9, and 81.4 ± 5.4%, respectively (*n*=4). After dissolution of extracted microstents in chloroform, no residual drug was found. Slow *in vitro* drug release within a time frame of 5–6 weeks was found for PTX and CAPE LDD devices.On the contrary, PFD LDD devices showed fast *in vitro* drug release within 10 h (Figure 2B).

### Drug release *in vivo*

*In vivo*, slow drug release of PTX from LDD devices was found within a time frame of 12 weeks. For CAPE and PFD LDD devices, drug release was already completely finished at the first extraction date, 1 week postoperatively. The comparison of drug release *in vitro* and *in vivo* is shown in Figure 2C.

### Histology

**Figure 3:**
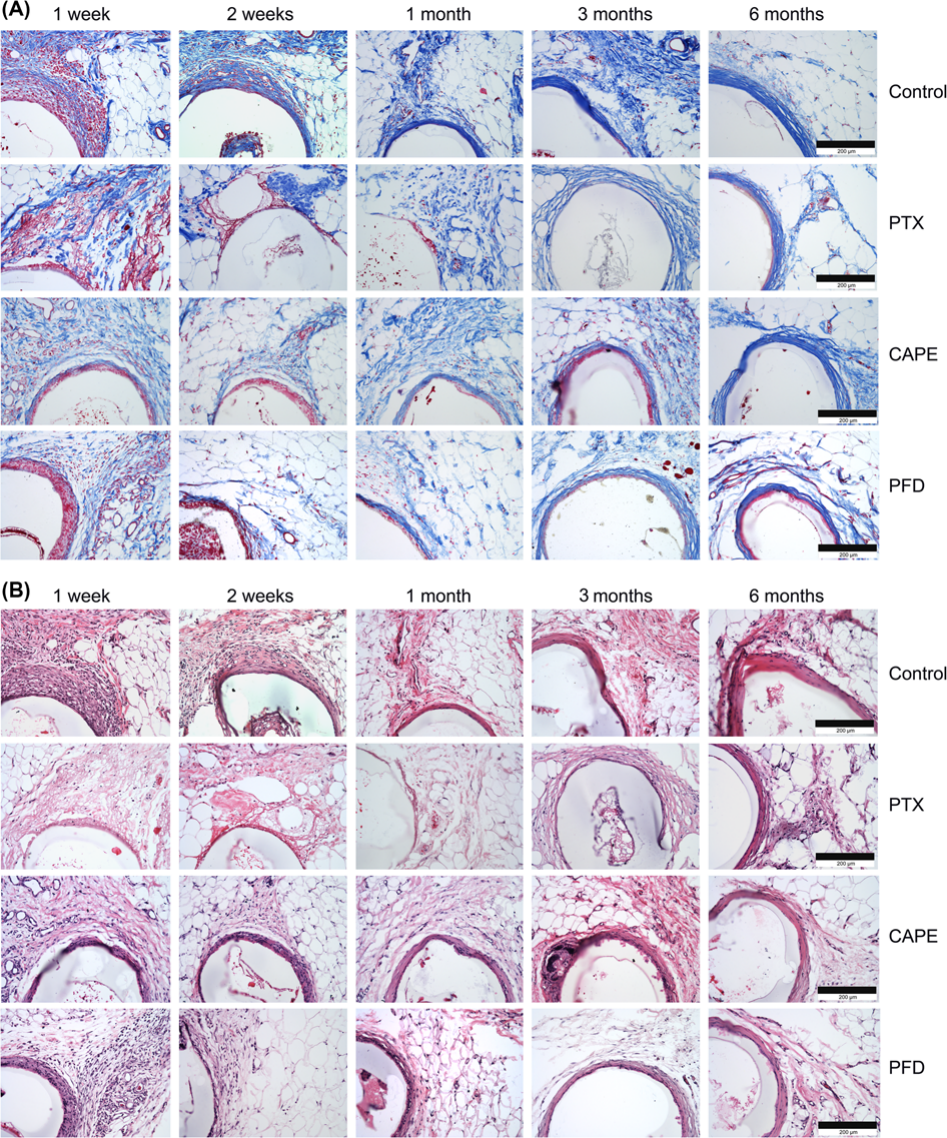
Histology of the subcutaneous white fat depots including the test specimens (**A**) Cross-sections of the rat white fat depots were stained for connective tissue using AZAN staining. A connective tissue-rich fibrotic capsule is obvious after 6 months in the periphery of uncoated control implants, as well as in the PTX-, CAPE-, and PFD-coated specimens. (**B**) Cross-sections were H&E stained. Comparable with (A), strongest implant encapsulation is obvious after 6 months. Bars represent 200 µm.

Histological examination of explanted fat depots including the test specimen implants revealed a massive cell infiltration around uncoated control, and coated CAPE and PFD test specimens 1–2 weeks after the implantation (Figure 3A,B). In contrast, around the PTX-coated test specimen a breakdown/damage of connective and fat tissues was detectable at these time points. Total 1 month after implantation, compact capsules around the control implants could be observed, which were also present in CAPE and PFD test specimens but not as compact as in controls. The fibrotic capsules increased in thickness up to 6 months in these groups. At this time point, some degradation of the biodegradable stent material was observed, which still formed a functional tube and was explantable for drug recovery examinations.

**Figure 4:**
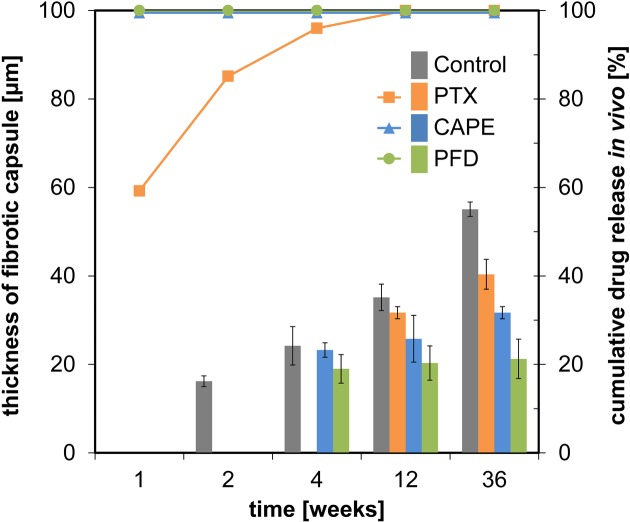
Evaluation of the fibrotic tissue response in comparison with drug release data *in vivo* Histological sections (Figure 3A) were used to measure the thickness of fibrotic capsules (left *y*-axis) surrounding uncoated and drug-coated test specimens. Results are depicted as colored bars (*n*=3; mean ± S.D.). Drug release data of coated test specimens (right *y*-axis), as determined with HPLC, are depicted as curves in corresponding colors (*n*=4, mean). After 1 week no residual drug could be quantitated for CAPE- and PFD-coated test specimens. Fibrotic tissue reactions for these two coatings were visible after 4 weeks, whereas PTX-coated test specimens showed fibrotic capsules after 12 weeks. In uncoated samples, fibrotic capsules were observed starting 2 weeks after implantation. Mean ± S.D. (each *n*=3).

Total 3 months after implantation, a fibrotic capsule had formed around the PTX test specimens as well. The fibrotic connective tissue was loosely packed and not as compact as in uncoated controls and CAPE- and PFD-coated specimens (Figure 3A,B). However, after 6 months, a compact fibrotic capsule could also be observed around the PTX-coated specimens. A comparison of capsular thicknesses in all groups revealed the thickest fibrotic capsules in the control test specimen group (Figure 4). The fibrotic capsule surrounding the PFD-coated specimens were least pronounced with only a minimal increase in thickness throughout the observation period (Figure 4).

**Figure 5:**
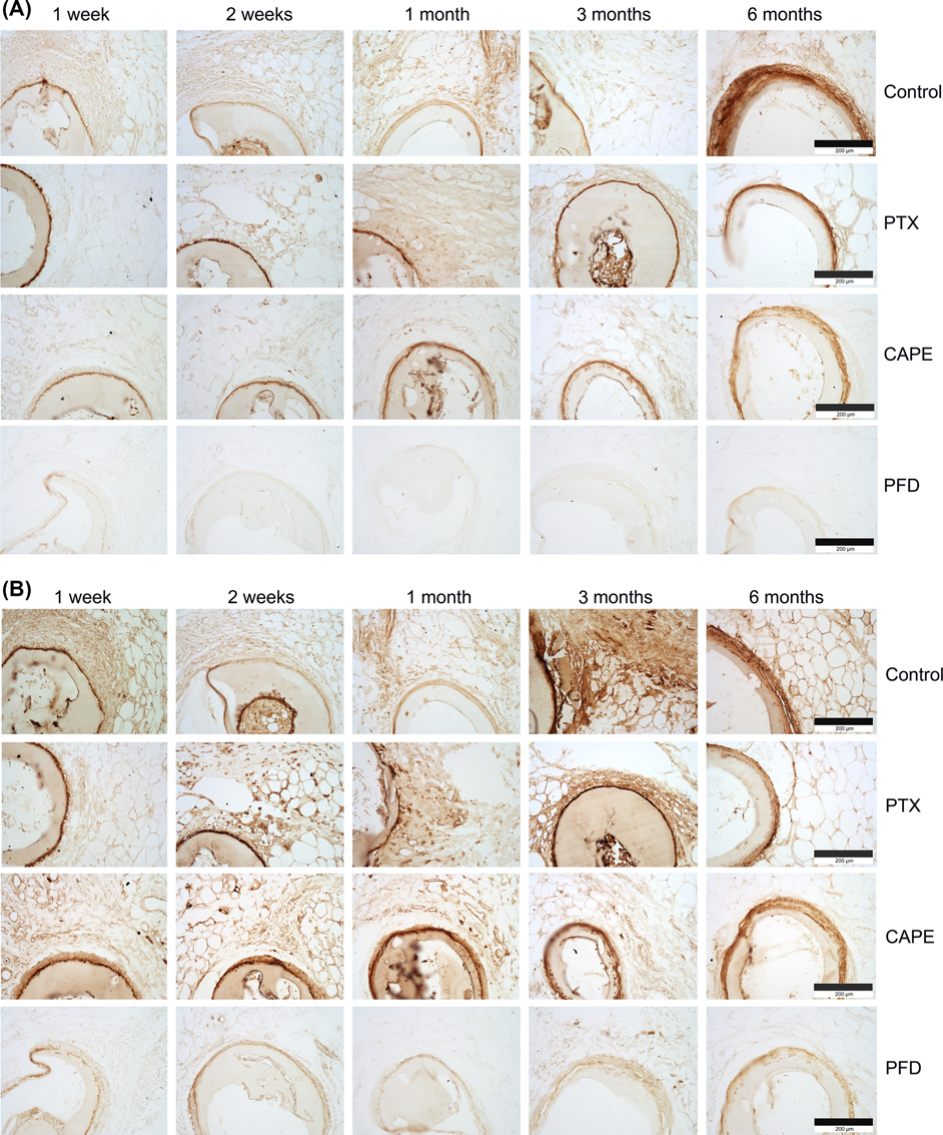
Immunohistochemical evaluation of the fibrotic tissue response to test specimens (**A**) The cross-sections of the rat fat depots including the test specimens were stained for collagen I. The connective tissue-rich fibrotic capsule is positively stained for collagen I with lowest expression in the PFD group. (**B**) Immunohistochemical examinations of cross-sections stained for collagen VI revealed high reactivity of the connective tissue-rich fibrotic capsules, which ensheath the implants with lowest reactivity in the PFD group. Bars represent 200 µm.

**Figure 6:**
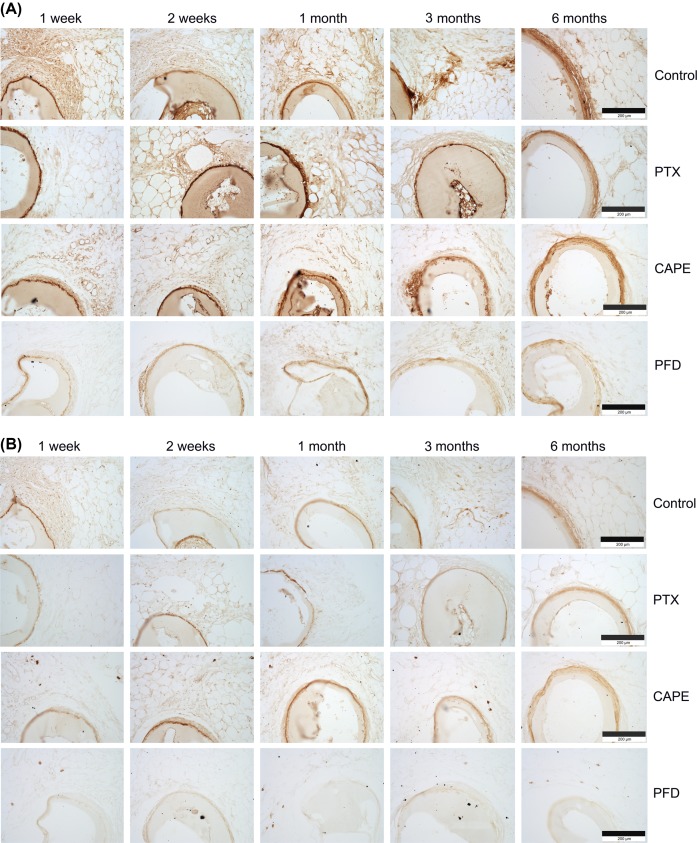
Immunohistochemistry of the subcutaneous white fat depots including the test specimen implants (**A**) Immunohistochemical reactivity against fibronectin could be detected after 6 months around the uncoated control and the drug-coated implants with weakest signal in the PFD group. (**B**) The cross-sections of the rat fat depots including the test specimens were stained for CD11b. Expression could be detected around the uncoated control and the drug-coated implants. Bars represent 200 µm.

Immunohistochemical examination of the fat depots including the test specimen implants showed positive signals against members of the ECM in the surrounding tissues and the compact fibrotic capsules. The capsules stained positive for collagen I (Figure 5A) and collagen VI (Figure 5B). Additionally, the compact fibrotic capsules stained positively for the ECM component fibronectin (Figure 6A). The strongest reactivity against ECM components could be detected in uncoated test specimens (control) in comparison with the other groups. It is slightly less pronounced in drug-coated test specimen (CAPE), and more obvious in PTX and PFD test specimens. Weakest reactivity against ECM members was detected in PFD-coated test specimens, confirming the results of the standard histological examinations, where thinnest fibrotic capsules were observed in the PFD test specimen group.

Examinations of the immune response following test specimen implantation were carried out using an antibody directed against the antigen CD11b, which is expressed by monocytes, macrophages, and dendritic cells. Positive inflammatory cells could be detected in the periphery of the test specimen in uncoated control and CAPE- and PFD-coated implants after 1 week (Figure 6B). Inflammatory cells could be identified in these groups at time points up to 6 months. In contrast, around PTX-coated implants inflammatory cells could be detected only 2 weeks postsurgery.

## Discussion

Prevention of scarring and fibrotic processes represents one of the most prominent challenges in fistulating glaucoma surgery. To overcome fibrosis, which leads to the failure of surgically created draining routes or occlusion of draining implants [41,35], modulating the postoperative fibrotic response with antifibrotic drugs has been a strategy in the recent past. In this context the cytostatics mitomycin C and 5-fluorouracil [25], which inhibit mitotic processes, have been used as antifibrotic agents. Especially, the use of mitomycin C has improved the success rate in fistulating glaucoma surgery, but its associated failure rate of up to 45% 5 years postoperatively is high [42,26] and its use is often coupled with side effects leading to sight-threatening complications like bleb leakage or blebitis, hypotony, choroidal detachment, and corneal impairment [43–45]. The inhibitor PTX, which inhibits mitosis by irreversible binding to the microtubule cytoskeleton, also seems to be a promising drug to inhibit postoperative fibrosis [26,27]. However, consistent with its mechanism of action, PTX affects every cell as an unspecific drug. In the last years, the possibility to incorporate antifibrotic drugs into GDDs and use them as drug-delivery systems has been investigated to prevent scarring or fibrotic encapsulation of the implant [25,46]. This approach seems to deliver the opportunity for a slow-release mechanism of antifibrotic drugs in low concentrations over a long period of time directly at the targetted tissue site. In a recent example, it was shown that long-term mitomycin C release could be measured up to 3 months postoperatively but was associated with retinal impairment in rabbit models *in vivo* [25,46]. Hydrogels and non-degradable polymers as well as biodegradable polymers have been used as antifibrotic drug delivery systems in animal studies [25,47,48].

Here, we investigated the fibrotic response to the implantation of test specimens made of a blend of two biodegradable hydroxybutyric acid-based polymers (P(4HB)/at.P(3HB)) 50/50% (w/w) into the white fat depots of rats. This polymer blend is characterized by low cytotoxicity and good *in vivo* biocompatibility [27]. The test specimens were uncoated (controls) or coated with the unspecific cytostatic drug PTX and the more specific antifibrotic drugs CAPE and PFD.

The manufactured test specimens yielded reproducible quality in terms of morphology, initial drug loading and drug recovery after processing. Nevertheless, for PTX and CAPE groups a more porous surface morphology was found when compared with the PFD group. This is probably due to different crystallinities of the used drugs. Irrespective of the drug used, an initial loading and recovery of 20 µg and 76% was found, respectively.

Within the present study, we could show distinct differences in drug release behavior between the *in vitro* and *in vivo* conditions, and the different drugs PTX, CAPE, and PFD. Due to the relatively slow degradation of the used semicrystalline P(4HB) within up to 52 weeks, we assume that drug release primary is diffusion controlled [49]. Nevertheless, our own yet unpublished *in vitro* degradation studies with different P(4HB)/at.P(3HB) blends suggests, that degradation accelerates with increasing content of amorphous at.P(3HB) and therefore, might play a minor role in the current case.

Due to poor solubility of PTX and CAPE in aqueous solutions, *in vitro* drug release profiles are relatively slow compared with PFD. The solubility of PFD in aqueous solutions is approximately five orders of magnitude higher (20.000 mg.l^−1^), compared with PTX (0.3 mg.l^−1^), exemplary [50,51]. Therefore, *in vitro* drug release of PFD is considerably faster (Figure 2B).

*In vivo* another effect predominates the drug release kinetics. Here, PTX release is decelerated compared with CAPE and PFD and compared with the *in vitro* data of PTX release (Figure 2C). The molecular weight of PTX (853.9 g mol^−1^) is greater by factors 3–4.6 than CAPE (284.1 g mol^−1^) and PFD (185.2 g mol^−1^) and appears to be the dominating parameter, decelerating diffusion controlled drug release of PTX *in vivo* (Sigma–Aldrich Corp. 2017). PTX release *in vivo* may be also decelerated due to its lower lipid solubility and saturation effects and therefore, delayed drug diffusion inside the rat fat tissue. Differences in *in vitro* and *in vivo* release behavior possibly may be influenced through different drug crystallinity. Kind of solvent and drying conditions like time and temperature within the drug-coating process are significant for the drug crystallinity. This may be an explanation for the different morphologies shown in Figure 2A.

Fibrotic encapsulation following GDD implantation is a known process and one of the most important reasons for implant failure [52,53]. In contrast, no fibrotic response was observed in rabbits after 38 weeks *in vivo* following implantation of biologically derived hydroxybutyric acid based polymers in our previous studies [27,35]. Here, for uncoated implants based on polymer blend of the biologically derived P(4HB) and the synthetic at.P(3HB) 50/50% (w/w), we could detect a massive cell infiltration 1 week after implantation in the periphery of the implant. These cells seem to ensheath the implants and start to synthesize ECM components leading to the formation of a fibrotic capsule and total implant-encapsulation 2–4 weeks after implantation. These fibrotic capsules increased in thickness up to 6 months. One difference between the abovementioned studies to our examinations is the implantation into white fat depots in rats, which could have an influence to the wound-healing process and the fibrotic response to implants. It has been reported that mature adipocytes repopulate skin wounds, adipocyte precursor cells proliferate, and that adipocytes as a key player of the intercellular communication are necessary for fibroblast recruitment and function in mice [54]. Additionally, wound-healing processes are different between rabbits and rats as shown by Kim et al. [55]. Both circumstances may explain observed prominent fibrosis in white fat depots in rats in comparison with Tenon’s – and suprachoroidal space in rabbit models. These findings support the suitability of the rat model for fibrotic investigations due to fibrotic implant encapsulation at similar intervals in humans.

The formation of a fibrotic capsule around the implant could be delayed by the antifibrotic drug PTX in our examinations. In early postoperative stages (1–2 weeks), a disturbance in the organization of connective and fat tissues and lesions peripherally to the PTX-coated test specimens were obvious. The appearance of lesions and skin ulcers has been described after intradermal injections of PTX in a mouse model [56], and degenerative and necrotic changes in the form of cytoplasmic vacuolization, fatty change, and apoptosis has been observed in a mammary tumor model in rats [57]. However, encapsulation comparable with the other groups and ill-defined fat tissue with abundant adipocytes could be detected after 6 months in our study.

Test specimens coated with CAPE showed only negligible antifibrotic effects. CAPE has been described with a broad spectrum of biological activities, including anti-inflammatory, antifibrotic, antioxidant, and immune-modulatory properties [58]. Mia and Bank [59] demonstrated an antifibrotic effect of CAPE in human dermal and lung fibroblasts, where an inhibition of fibroblast transformation into myofibroblasts and a suppression of collagen and fibronectin expression were shown. In other studies, the antifibrotic effects of CAPE led to reduced collagen and fibronectin deposition in mice and rats *in vivo* [28,60,61].

The same applies for PFD, where *in vitro* studies demonstrated inhibition of cell proliferation, myofibroblast differentiation, collagen contractility, cytokine suppression, decreased collagen and fibronectin expression, and migratory ability of cardiac, renal, lung, hepatic, and ocular fibroblasts [31,32,62–66]. An antifibrotic effect *in vivo* has also been described in a trabeculectomy model in rabbits, where postoperative application of PFD was associated with less scarring and improved bleb survival [67] and following GDI implantation in a rabbit model [68]. In our examination, neither PFD nor CAPE completely prevented fibrosis and implant encapsulation in the white fat depot in rats, but an attenuation of fibrosis was observed in the PFD group, characterized by a reduction in fibrotic capsule thickness (Figure 4), compaction, and immunohistochemical reactivity against examined ECM components.

Based on the presented data, the influence of the surface morphology of the test specimens on fibrotic encapsulation behavior could not be clearly distinguished. However, it is obvious that the use of an antifibrotic drug (i.e. PFD) retards and reduces fibrotic encapsulation compared with the control group with the same surface morphology as the PFD group but with no drug incorporated (Figure 4). It can be speculated that fibrotic encapsulation in the PTX and CAPE groups would also be further retarded and reduced, for a more similar surface morphology to the PFD group.

In wound-healing processes, directly after blood clotting and vasoconstriction, the infiltration of leukocytes and an immune response is initiated [69]. Most immune competent cells are characterized by the expression of the surface antigen CD11b, a member of the integrin family, which is expressed on the surface of leukocytes including monocytes, neutrophils, natural killer cells, granulocytes, and macrophages [70]. In our examinations, we observed an infiltration of immune competent cells in the periphery of uncoated control test specimens as well as around CAPE- and PFD-coated implants after 1 week, which also could be detected up to 6 months in moderate amounts. Other studies revealed an absence of inflammation following suprachoroidal shunt implantation after 15 weeks [71]. We suggest that an ongoing attraction of immune competent cells is triggered by the biodegradation process of the polymer blend and cytokine secretion from activated myofibroblasts [72] also after the acute phase in wound healing. However, in the periphery of PTX-coated implants inflammatory cells were only detectable 2 weeks postsurgically. This finding is comparable with a study of the body reaction in glaucoma drainage implant surgery, in which a peak of inflammation was observed 2 weeks postoperatively [73].

To summarize our findings, postoperative fibrotic and immune responses can be attenuated by glaucoma implants coated with antifibrotic drugs. The management of controlled drug release by avoiding a burst release to guarantee a long-term medication can increase the pharmaceutical effects of antifibrotic drugs with low risk of side effects to sensitive ocular structures. As a further developmental step, the combination of different antifibrotic pharmaceuticals is also conceivable to potentially increase effectiveness. Further investigations will help to develop GDDs with drug delivery characteristics to create a permanent drainage path to lower IOP and prevent glaucoma progression.

## Conclusion

Implantation of drug-coated test specimens into the subcutaneous white fat depot in rats opens up new possibilities to investigate the release kinetics of antifibrotic agents *in vivo*, which can be compared with the retrobulbar fat depot in humans. The antifibrotic potential as well as drug-induced side effects were analyzed by histological examinations. PTX delayed the formation of a fibrotic capsule until 12 weeks after implantation. However, the PTX coating caused side effects and could not maintain its antifibrotic activity over the entire observation period of 6 months. The agents CAPE and PFD delayed the formation of a fibrotic capsule around the implants by 2 weeks in comparison with the control group. The PFD coating was overall most effective with regard to capsule thickness and growth of the capsule throughout the observation period.

In conclusion, these investigations help to identify new antifibrotic agents,which can be used as medicinal drug coatings for microstents in the treatment of glaucoma draining aqueous humor into the intra-orbital fat depot or tenon space. Our examinations allow the analysis of side effects and the quantitation of drug-specific antifibrotic potential.

## Abbreviations

at.P(3HB): atactic poly(3-hydroxybutyrate)
AZAN: azocarmine G- aniline blue - orange G staining
CAPE: caffeic acid phenethyl ester
ECM: extracellular matrix
GDD: glaucoma drainage device
GDI: glaucoma drainage implant
IHC: immunohistochemistry
IOP: intraocular pressure
LDD: local drug delivery
PFA: paraformaldehyde
PFD: pirfenidone
PTX: paclitaxel
P(4HB): poly(4-hydroxybutyrate)
SEM: scanning electron microscope
TGF: transforming growth factor
TNF: tumor necrosis factor

## References

[1] Weinreb R.N., Aung T. and Medeiros F.A. (2014) The pathophysiology and treatment of glaucoma: a review. JAMA 311, 1901–1911 10.1001/jama.2014.3192 24825645 PMC4523637

[2] Kass M.A. (2002) The ocular hypertension treatment study: a randomized trial determines that topical ocular hypotensive medication delays or prevents the onset of primary open-angle glaucoma. Arch. Ophthalmol. 120, 701–713 10.1001/archopht.120.6.701 12049574

[3] Sambhara D. and Aref A.A. (2014) Glaucoma management: relative value and place in therapy of available drug treatments. Ther. Adv. Chronic Dis. 5, 30–43 10.1177/2040622313511286 24381726 PMC3871276

[4] Remo S.J. and Wang-Pui S. (2004) Comparison of latanoprost with fixed combination dorzolamide and timolol in adult patients with elevated intraocular pressure: an eight week, randomized, open-label, parallel-group, multicenter study in Latin America. Clin. Ther. 26, 755–756 10.1016/S0149-2918(04)90075-6 15220019

[5] Moisseiev E., Kurtz S., Lazar M. and Shemesh G. (2013) Intraocular pressure reduction using a fixed combination of timolol maleate 0.5% and brimonidine tartrate 0.2% administered three times daily. Clin. Ophthalmol. 7, 1269–1273 10.2147/OPTH.S47760 23836956 PMC3699296

[6] Cairns J.E. (1968) Trabeculectomy. Preliminary report of a new method. Am. J. Ophthalmol. 66, 673–679 10.1016/0002-9394(68)91288-9 4891876

[7] Fedorov S.N., Ioffe D.I. and Ronkina T.I. (1982) Glaucoma surgery – deep sclerectomy. Vestn. Ophthal. 4, 6–10 7135737

[8] Lim K.S., Allan B.D., Lloyd A.W., Muir A. and Khaw P.T. (1998) Glaucoma drainage devices; past present, and future. Br. J. Ophthalmol. 82, 1083–1089 10.1136/bjo.82.9.1083 9893602 PMC1722728

[9] Noureddin B.N., Zein W., Haddad C., Ma’luf R. and Bashshur Z. (2006) Diode laser transcleral cyclophotocoagulation for refractory glaucoma: a 1 year follow-up of patients treated using an aggressive protocol. Eye 20, 329–335 10.1038/sj.eye.6701875 15877101

[10] Kaplowitz K., Kuei A., Klenofsky B., Abazari A. and Honkanen R. (2015) The use of endoscopic cyclophotocoagulation for moderate to advanced glaucoma. Acta Ophthalmol. 93, 395–401 10.1111/aos.12529 25123160

[11] Molteno A. (1969) New implant for drainage in glaucoma. Clinical trial. Br. J. Ophthalmol. 53, 606–615 10.1136/bjo.53.9.606 4900144 PMC1207524

[12] Prata J.A. Jr, Minckler D.S., Mermoud A. and Baerveldt G. (1996) Effects of mitomycin-C on the function of Baerveldt glaucoma drainage implants in rabbits. Glaucoma 5, 29–38 10.1097/00061198-199602000-00006 8795731

[13] Allemann R. (2011) Ultra high-field magnetic resonance imaging of a glaucoma microstent. Curr. Eye Res. 36, 719–726 10.3109/02713683.2011.587936 21780921

[14] Guthoff R.F. (2009) Development of a glaucoma microstent with drainage into the suprachoroidal space: fluid mechanical model approach. Ophthalmology 106, 805–812 10.1007/s00347-009-1929-x 19806382

[15] Schmidt W. (2010) Konzept eines druckgesteuerten Mikrostents für die Glaukomtherapie. Concept of a pressure-controlled microstent for glaucoma therapy. Klin. Monbl. Augenheilkd. 227, 946–952 10.1055/s-0029-1245928 21157664

[16] Dietlein T.S., Jordan J., Lueke C. and Krieglstein G.K. (2008) Modern concepts in antiglaucomatous implant surgery. Graef. Arch. Clin. Exp. Ophthalmol. 246, 1653–1664 10.1007/s00417-008-0899-z 18682974

[17] Löbler M. (2013) Ocular fibroblast types differ in their mRNA profiles-implications for fibrosis prevention after aqueous shunt implantation. Mol. Vis. 19, 1321–1331 23805039 PMC3692408

[18] Stahnke T. (2012) Different fibroblast subpopulations of the eye: a therapeutic target to prevent postoperative fibrosis in glaucoma therapy. Exp. Eye Res. 100, 88–97 10.1016/j.exer.2012.04.015 22579993

[19] Møller P.M. (1955) Chapter VII: Orbital pressure in normal subjects (with statistical calculation of the results). Acta Ophthalmol. 53–56, 10.1111/j.1755-3768.1955.tb00096.x13410568

[20] Otto A.J., Koornneef L., Mourits M.P. and van Leeuwen L.D. (1996) Retrobulbar pressures measured during surgical decompression of the orbit. Br. J. Ophthalmol. 80, 1042–1045 10.1136/bjo.80.12.1042 9059266 PMC505699

[21] Xie X. (2013) Noninvasive intracranial pressure estimation by orbital subarachnoid space measurement: the Beijing Intracranial and Intraocular Pressure (iCOP) study. Crit. Care 17, R162 10.1186/cc12841 23883736 PMC4056099

[22] Cinti S. (2005) The adipose organ. Prostaglandins Leukoc. Essent. Fatty Acids 73, 9–15 10.1016/j.plefa.2005.04.010 15936182

[23] Bielen R., Bennett J., Ferdinande B. and Dubois C. (2014) Drug-eluting versus bare metal stents after rotational atherectomy: clinical outcome in a single centre. Acta Cardiol. 69, 611–617 10.1080/AC.69.6.1000003 25643431

[24] Windecker S. (2015) Comparison of a novel biodegradable polymer sirolimus-eluting stent with a durable polymer everolimus-eluting stent: results of the randomized BIOFLOW-II Trial. Circ. Cardiovasc. Interv. 8, e001441 10.1161/CIRCINTERVENTIONS.114.001441 25634905

[25] Mostafaei A. (2011) Augmenting trabeculectomy in glaucoma with subconjunctival mitomycin C versus subconjunctival 5-fluorouracil: a randomized clinical trial. Clin. Ophthalmol. 5, 491–494 10.2147/OPTH.S17328 21573097 PMC3090304

[26] Choritz L., Grub J., Wegner M., Pfeiffer N. and Thieme H. (2010) Paclitaxel inhibits growth, migration and collagen production of human Tenon’s fibroblasts - potential use in drug-eluting glaucoma drainage devices. Graef. Arch. Clin. Exp. Ophthalmol. 248, 197–206 10.1007/s00417-009-1221-4 19898860 PMC2801844

[27] Löbler M. (2011) Polymers and drugs suitable for the development of a drug delivery drainage system in glaucoma surgery. J. Biomed. Mater. Res. B Appl. Biomater. 97, 388–395 10.1002/jbm.b.31826 21432996

[28] Larki-Harchegani A. (2013) Evaluation of the effects of caffeic acid phenethyl ester on prostaglandin E2 and two key cytokines involved in bleomycin-induced pulmonary fibrosis. Iran J. Basic Med. Sci. 16, 850–857 23997916 PMC3758057

[29] Larki A. (2013) Regulatory effect of caffeic acid phenethyl ester on type I collagen and interferon-gamma in bleomycin-induced pulmonary fibrosis in rat. Res. Pharm. Sci. 8, 243–252 24082893 PMC3757589

[30] Taylan M. (2016) The protective effects of caffeic acid phenethyl ester on acetylsalicylic acid-induced lung injury in rats. J. Invest. Surg. 16, 1–7 10.3109/08941939.2016.1149641 26980558

[31] Lin X., Yu M., Wu K., Yuan H. and Zhong H. (2009) Effects of pirfenidone on proliferation, migration, and collagen contraction of human tenon’s fibroblasts *in vitro*. Invest. Ophthalmol. Vis. Sci. 50, 3763–3770 10.1167/iovs.08-2815 19264889

[32] Stahnke T. (2017) Suppression of TGF-β pathway by pirfenidone decreases extracellular matrix deposition in ocular fibroblasts *in vitro*. PLoS ONE 12, e0172592 10.1371/journal.pone.0172592 28231275 PMC5322922

[33] Hilberg O., Simonsen U., du Bois R. and Bendstrup E. (2012) Pirfenidone: significant treatment effects in idiopathic pulmonary fibrosis. Clin. Respir. J. 6, 131–143 10.1111/j.1752-699X.2012.00302.x 22697264

[34] Hasdemir P.S. (2016) Effect of pirfenidone on vascular proliferation, inflammation and fibrosis in an abdominal adhesion rat model. J. Invest. Surg. 15, 1–7 10.1080/08941939.2016.1215578 27715339

[35] Hovakimyan M. (2015) Development of an experimental drug-eluting suprachoroidal microstent as glaucoma drainage device. Transl. Vis. Sci. Technol. 4, 10.1167/tvst.4.3.14 26175960 PMC4497488

[36] Jedlinski Z., Kowalczuk M., Glowkowski W., Grobelny J. and Szwarc M. (1991) Novel polymerization of β-butyrolactone initiated by potassium naphthalenide in the presence of a crown ether or a cryptand. Macromolecules 24, 349–352 10.1021/ma00002a002

[37] Hubbs J. (1997) Biodegradable poly(3-hydroxyalkanoate) compositions and blends. U.S. Pat. 5,625,029A

[38] Freier T. (2002) *In vitro* and *in vivo* degradation studies for development of a biodegradable patch based on poly(3-hydroxybutyrate). Biomaterials 23, 2649–2657 10.1016/S0142-9612(01)00405-7 12059014

[39] Mulisch M., Welsch U., (2010) In Romeis - Mikroskopische Technik, 18 edn., (Mulisch M., Welsch U., eds), Springer, Berlin, ISBN-10: 3827416760

[40] Stahnke T., Stadelmann C., Netzler A., Brück W. and Richter-Landsberg C. (2007) Differential upregulation of heme oxygenase-1 (HSP32) in glial cells after oxidative stress and in demyelinating disorders. J. Mol. Neurosci. 32, 25–37 10.1007/s12031-007-0005-8 17873285

[41] Thieme H. (2012) Current status of epibulbar antiglaucoma drainage devices in glaucoma surgery. Dtsch. Arztebl. Int. 109, 659–664 23094002 10.3238/arztebl.2012.0659 PMC3476613

[42] Giampani J. Jr, Borges-Giampani A.S., Carani J.C., Oltrogge E.W. and Susanna R. Jr (2008) Efficacy and safety of trabeculectomy with mitomycin C for childhood glaucoma: a study of results with long-term follow-up. Clinics (Sao Paulo) 63, 421–426 10.1590/S1807-59322008000400002 18719749 PMC2664114

[43] Masoumpour M.B., Nowroozzadeh M.H. and Razeghinejad M.R. (2016) Current and future techniques in wound healing modulation after glaucoma filtering surgeries. Open Ophthalmol. J. 10, 68–85 10.2174/1874364101610010068 27014389 PMC4780518

[44] Mearza A.A. and Aslanides I.M. (2007) Uses and complications of mitomycin C in ophthalmology. Expert. Opin. Drug Saf. 6, 27–32 10.1517/14740338.6.1.27 17181449

[45] van Bergen T., van de Velde S., Vandewalle E., Moons L. and Stalmans I. (2014) Improving patient outcomes following glaucoma surgery: state of the art and future perspectives. Clin. Ophthalmol. 8, 857–867 10.2147/OPTH.S48745 24833892 PMC4014365

[46] Sahiner N. (2009) Creation of a drug-coated glaucoma drainage device using polymer technology: *in vitro* and *in vivo* studies. Arch. Ophthalmol. 127, 448–453 10.1001/archophthalmol.2009.19 19365022

[47] Blake D.A. (2006) Inhibition of cell proliferation by mitomycin C incorporated into P(HEMA) hydrogels. J. Glaucoma 15, 291–298 10.1097/01.ijg.0000212236.96039.9c 16865005

[48] Polak M.B. (2008) Controlled delivery of 5-chlorouracil using poly(orthoesters) in filtering surgery for glaucoma. Invest. Ophthalmol. Vis. Sci. 49, 2993–3003 10.1167/iovs.07-0919 18579761

[49] Martin D.P. and Williams S.F. (2003) Medical applications of poly-4-hydroxybutyrate: a strong flexible ab-sorbable biomaterial. Biochem. Eng. J. 16, 97–105 10.1016/S1369-703X(03)00040-8

[50] Konno T., Watanabe J. and Ishihara K. (2003) Enhanced solubility of paclitaxel using water-soluble and biocompatible 2-methacryloyloxyethyl phosphorylcholine polymers. J. Biomed. Mater. Res. A 65, 209–214 10.1002/jbm.a.10481 12734814

[51] Macías-Barragán J., Sandoval-Rodríguez A., Navarro-Partida J. and Armendáriz-Borunda J. (2010) The multifaceted role of pirfenidone and its novel targets. Fibrogen. Tissue Rep. 3, 16 10.1186/1755-1536-3-16 PMC2944211 20809935

[52] Mahale A. (2015) Altered expression of fibrosis genes in capsules of failed Ahmed glaucoma valve implants. PLoS ONE 10, e0122409 10.1371/journal.pone.0122409 25879570 PMC4399875

[53] Quaranta L. (2016) Needle revision with 5-fluorouracil for the treatment of ahmed glaucoma valve filtering blebs: 5-fluoruracil needling revision can be a useful and safe tool in the management of failing ahmed glaucoma valve filtering blebs. J. Glaucoma 25, e367–e371 10.1097/IJG.0000000000000366 26766399

[54] Schmidt B.A. and Horsley V. (2013) Intradermal adipocytes mediate fibroblast recruitment during skin wound healing. Development 140, 1517–1527 10.1242/dev.087593 23482487 PMC3596993

[55] Kim D.J., Mustoe T. and Clark R.A. (2015) Cutaneous wound healing in aging small mammals: a systematic review. Wound Repair Regen. 23, 318–339 10.1111/wrr.12290 25817246

[56] Dorr R.T., Snead K. and Liddil J.D. (1996) Skin ulceration potential of paclitaxel in a mouse skin model *in vivo*. Cancer 78, 152–156 10.1002/(SICI)1097-0142(19960701)78:1%3c152::AID-CNCR21%3e3.0.CO;2-Y 8646711

[57] Al-Ghananeem A.M. (2009) Intratumoral delivery of paclitaxel in solid tumor from biodegradable hyaluronan nanoparticle formulations. AAPS Pharm. Sci. Tech. 10, 410–417 10.1208/s12249-009-9222-5 PMC2690785 19381833

[58] Murtaza G. (2014) Caffeic acid phenethyl ester and therapeutic potentials. Biomed. Res. Int. 145342, 10.1155/2014/145342 24971312 PMC4058104

[59] Mia M.M. and Bank R.A. (2016) The pro-fibrotic properties of transforming growth factor on human fibroblasts are counteracted by caffeic acid by inhibiting myofibroblast formation and collagen synthesis. Cell Tissue Res. 363, 775–789 10.1007/s00441-015-2285-6 26453399 PMC4761014

[60] Zhao W.X. (2014) Caffeic acid phenethyl ester attenuates pro-inflammatory and fibrogenic phenotypes of LPS-stimulated hepatic stellate cells through the inhibition of NF-кB signaling. Int. J. Mol. Med. 33, 687–694 10.3892/ijmm.2013.1613 24378685

[61] Chuang S.T., Kuo Y.H. and Su J. (2015) KS370G, a caffeamide derivative, attenuates unilateral obstruction-induced renal fibrosis by the reduction of inflammation and oxidative stress in mice. Eur. J. Pharmacol. 750, 1–7 10.1016/j.ejphar.2015.01.020 25620133

[62] Burghardt I. (2007) Pirfenidone inhibits TGF-beta expression in malignant glioma cells. Biochem. Biophys. Res. Commun. 354, 542–547 10.1016/j.bbrc.2007.01.012 17234158

[63] Shi Q. (2011) *In vitro* effects of pirfenidone on cardiac fibroblasts: proliferation, myofibroblast differentiation, migration and cytokine secretion. PLoS ONE 6, e28134 10.1371/journal.pone.0028134 22132230 PMC3223242

[64] Di Sario A. (2002) Effect of pirfenidone on rat hepatic stellate cell proliferation and collagen production. J. Hepatol. 37, 584–591 10.1016/S0168-8278(02)00245-3 12399223

[65] Hewitson T.D. (2001) Pirfenidone reduces in vitro rat renal fibroblast activation and mitogenesis. J. Nephrol. 14, 453–460 11783601

[66] Kim H. (2010) Antifibrotic effect of pirfenidone on orbital fibroblasts of patients with thyroid-associated ophthalmopathy by decreasing TIMP-1 and collagen levels. Invest. Ophthalmol. Vis. Sci. 51, 3061–3066 10.1167/iovs.09-4257 20053983

[67] Zhong H., Sun G., Lin X., Wu K. and Yu M. (2011) Evaluation of pirfenidone as a new postoperative antiscarring agent in experimental glaucoma surgery. Invest. Ophthalmol. Vis. Sci. 52, 3136–3142 10.1167/iovs.10-6240 21330661

[68] Jung K.I. and Park C.K. (2016) Pirfenidone inhibits fibrosis in foreign body reaction after glaucoma drainage device implantation. Drug Des. Dev. Ther. 10, 1477–1488 27143855 10.2147/DDDT.S99957 PMC4841429

[69] Engelhardt E. (1998) Chemokines IL-8, GROalpha, MCP-1, IP-10, and Mig are sequentially and differentially expressed during phase-specific infiltration of leukocyte subsets in human wound healing. Am. J. Pathol. 153, 1849–1860 10.1016/S0002-9440(10)65699-4 9846975 PMC1866330

[70] Schmid M., Wege A.K. and Ritter U. (2012) Characteristics of “Tip-DCs and MDSCs” and their potential role in leishmaniasis. Front. Microbiol. 3, 74 22416241 10.3389/fmicb.2012.00074 PMC3298847

[71] Oatts J.T. (2013) *In vitro* and *in vivo* comparison of two suprachoroidal shunts. Invest. Ophthalmol. Vis. Sci. 54, 5416–5423 10.1167/iovs.13-11853 23847318 PMC4141703

[72] Rogler G. (2001) Differential activation of cytokine secretion in primary human colonic fibroblast/myofibroblast cultures. Scand. J. Gastroenterol. 36, 389–398 10.1080/003655201300051216 11336164

[73] Jung K.I., Lee S.B., Kim J.H. and Park C.K. (2013) Foreign body reaction in glaucoma drainage implant surgery. Invest. Ophthalmol. Vis. Sci. 54, 3957–3964 10.1167/iovs.12-11310 23674756

